# Supplementary material for: Impact of RMB internationalization on China’s competitiveness in financial services trade based on the VAR model: Evidence from China-US
Source: PLoS One. 2024 Jun 4;19(6):e0302845. doi: 10.1371/journal.pone.0302845 (PMC11149869; doi:10.1371/journal.pone.0302845)
Supplement: Attachment — Submitted filename: Comments on PONE-D-23-42358.docx [file pone.0302845.s002.docx]

**Comments on**

**Impact of RMB Internationalization on China’s Competitiveness in Financial Services Trade based on VAR Model: Evidence from China-US**

Recommendation: Major revision

1. Author applies the VAR model to explore the potential impact of RMB internationalization on China’s competitiveness in China-US financial services trade. It is an interesting perspective to study RMB internationalization and its impacts.
2. Other determining factors of financial service trade other than RMB internationalization should be considered and included in the model as control variables.
3. Robustness test can be conducted by using other indicators of RMB internationalization measurement. It will improve reliability of empirical results.
4. Multicollinearity should be carefully considered in author’s VAR model since it is highly possible that there is multilinear among variables on level of RMB internationalization.
5. Minor errors: 4.1 and 4.2 titles are the same; English writing could be improved by academic editing.
