# Supplementary material for: Impact of RMB internationalization on China’s competitiveness in financial services trade based on the VAR model: Evidence from China-US
Source: PLoS One. 2024 Jun 4;19(6):e0302845. doi: 10.1371/journal.pone.0302845 (PMC11149869; doi:10.1371/journal.pone.0302845)
Supplement: Attachment — Submitted filename: Response to Reviews.docx [file pone.0302845.s003.docx]

# Response letter

## Title: Impact of RMB Internationalization on China's Competitiveness in Financial Services Trade based on VAR model: Evidence from China-US

## Number.: PONE-D-23-42358

I express my profound gratitude to the esteemed editors and two diligent reviewers for their invaluable contributions in the form of constructive comments and suggestions. Their insightful feedback has played a pivotal role in improving the overall quality and scholarly merit of my manuscript. I have meticulously addressed each comment and suggestion in a detailed and methodical manner, offering comprehensive responses in the section below. Additionally, for your easy reference, all the main amendments in the revised paper are highlighted in yellow. Furthermore, the comments and recommendations provided by the editors and reviewers have been distinctly presented in italics, enabling effortless comprehension and reference.

**Response to the Editor:**

**Editor point #1:** *Please ensure that your manuscript meets PLOS ONE's style requirements, including those for file naming. The PLOS ONE style templates can be found at：*

*https://journals.plos.org/plosone/s/file?id=wjVg/PLOSOne_formatting_sample_main_body.pdf and*

*https://journals.plos.org/plosone/s/file?id=ba62/PLOSOne_formatting_sample_title_authors_affiliations.pdf*

**Author response #1:** I sincerely appreciate your valuable comments and suggestions provided for my manuscript. I have thoroughly reviewed my manuscript and meticulously updated the references in accordance with the prescribed Numbered reference style adopted by *PLOS ONE*. This rigorous adherence to the journal’s guidelines not only ensures compliance but also promotes consistency in the format and style across the entire manuscript.

**Editor point #2:** *We suggest you thoroughly copyedit your manuscript for language usage, spelling, and grammar. If you do not know anyone who can help you do this, you may wish to consider employing a professional scientific editing service.*

*Whilst you may use any professional scientific editing service of your choice, PLOS has partnered with both American Journal Experts (AJE) and Editage to provide discounted services to PLOS authors. Both organizations have experience helping authors meet PLOS guidelines and can provide language editing, translation, manuscript formatting, and figure formatting to ensure your manuscript meets our submission guidelines. To take advantage of our partnership with AJE, visit the AJE website (http://learn.aje.com/plos/) for a 15% discount off AJE services. To take advantage of our partnership with Editage, visit the Editage website (www.editage.com) and enter referral code PLOSEDIT for a 15% discount off Editage services. If the PLOS editorial team finds any language issues in text that either AJE or Editage has edited, the service provider will re-edit the text for free.*

**Author response #2:** I extend my sincere gratitude for your valuable feedback regarding my manuscript. In response to your insightful recommendations, I have taken the initiative to utilize the esteemed Editage(www.editage.com)’s Language Editing services, having placed an order with No.IJNRO_1. Details of this professional service and a copy of the manuscript with using track changes or highlighting have been uploaded as a zip file in Supporting Documents. Furthermore, I have diligently revised the manuscript, paying particular attention to rectifying any grammatical errors and enhancing the overall quality of the language used. Rest assured, the revised manuscript now aligns with the stringent standards set by *PLOS ONE*. I am confident that my efforts to improve the language quality will meet your expectations.

**Editor point #3:** *Please include captions for your Supporting Information files at the end of your manuscript, and update any in-text citations to match accordingly. Please see our Supporting Information guidelines for more information: http://journals.plos.org/plosone/s/supporting-information.*

**Author response #3:** Your suggestions have been immensely helpful in improving the quality and normativeness of my research. Taking into consideration your suggestion, I have incorporated a dedicated section titled “Support Information” (on page 35) right before the “References” section. The Supporting Information includes one dataset (hyperlink) containing all the data required for the models and tests of this article (click the hyperlink to open the Excel sheet). And, I have uploaded this data named "New Dataset" in the Supporting Information. The data availability statement is as follows: Data will be made accessible upon request. Besides, I have uploaded all the figures in the article to PACE.

In order to improve the readability of the article and ensure standardization, I carefully reviewed the citations in the article and made necessary adjustments and updates to the references based on your insightful suggestions.

**Response to Reviewer 1:**

**Reviewer point #1:** *Author applies the VAR model to explore the potential impact of RMB internationalization on China’s competitiveness in China-US financial services trade. It is an interesting perspective to study RMB internationalization and its impacts.*

**Author response #1:** I really appreciate your interest and comments on my research. This article is dedicated to finding ways for the Chinese government to improve the competitiveness of financial services trade in the context of RMB internationalization. I believe this research motivation is meaningful and necessary.

**Reviewer point #2:** *Other determining factors of financial service trade other than RMB internationalization should be considered and included in the model as control variables.*

**Author response #2:** I appreciate your insightful advice. Through further study of relevant literature[1][2] and in-depth analysis of the research content, I admit that this study should select other influencing factors of financial services trade as control variables and add them to the model.

Following Xiang[3], this study considers the availability of data and the loss of a certain degree of freedom due to the length of the lag period in the VAR system. Thus, the following three control variables are added to the model. First, the level of economic development, measured by the proportion of China's GDP to the world's total GDP (GDPCG). A good economic foundation is a prerequisite for the development of financial services trade. The higher the level of economic development of a country, the stronger the competitiveness of its financial services trade. Second, the level of development of the financial industry, measured by the proportion of the added value of China's financial industry to its GDP (FGDPC). Financial services trade between countries often needs to be realized through financial institutions. The high-quality development of the financial industry can effectively improve the competitiveness of the financial services trade. Third, the scale of goods trade, measured by the growth rate of China's total imports and exports of goods trade (GTR). Financial services trade is inseparable from goods trade, which often drives the development of related financial services trade.

Through the ADF method, all the above control variables passed the stationarity test. The overall model remains stable after adding new control variables and the empirical results are consistent with the theoretical analysis. This approach can make the var model analyze the relationship between independent variables and dependent variables more comprehensively and accurately, and improve the reliability of research conclusions.

**References**

[1] Bernanke, B. S. Alternative explanations of the money-income correlation. In Carnegie-Rochester Conference Series on Public Policy. 1986;25:49-99 [https://doi.org/10.3386/w1842](https://doi.org/10.3386/w1842)
[2] Blanchard, O. J., & Quah, D. The dynamic effects of aggregate demand and supply disturbances.The American Economic Review. 1989;79:655-673. <https://doi.org/10.3386/w2737>
[3] [Xiang YX, Wang XM. Research on the impact of currency internationalization on the competitiveness of financial services trade. Western Forum. 2022;04:41-54.](https://kns.cnki.net/kcms2/article/abstract?v=M7N75Hb03FX4ON-Xgymo4KQ6E7Pf-0PK-UrHtfabjOcTPK_db4PsZ4KJOq6Z4xbuBDiUFiveca3SSYO66pNTeb0oFJUDVNaWB02o5LE9wo_8Un11ztgTkqX-j2WBWuxT55vtBREEqP-8fInPp6-NPw==&uniplatform=NZKPT&language=CHS)

**Reviewer point #3:** *Robustness test can be conducted by using other indicators of RMB internationalization measurement. It will improve reliability of empirical results.*

**Author response #3:** I would like to express my gratitude for your insightful comments. To ensure the robustness of the conclusions, this study replaces the different measurement indicators of RMB internationalization and again performs the impulse response analysis (on page 32). The International Monetary Research Institute of Renmin University of China posits that with the orderly opening of the RMB capital account, the international monetary function of the RMB should be better reflected in the real economy. Specifically, it emphasizes the functions of the RMB as a currency for trade pricing and settlement, direct investment, and international bond transactions, and uses this to select appropriate variables and indicators to compile a comprehensive multivariable composite index, the RII. This is used to measure and reflect the level of RMB internationalization. Meanwhile, the PBOC has compiled the Cross-border RMB Index (CRI) by tracking the active use of the RMB in three key links: cross-border outflows, overseas transfers, and cross-border returns. This index transmits information on cross-border and overseas use and circulation of the RMB to customers in a more intuitive way, and more comprehensively reflects the development of RMB internationalization.

These two indicators are relatively authoritative and comprehensive indicators for measuring the degree of RMB internationalization; therefore, they are selected for robustness testing. It can be seen from the impulse response analysis that the impact effects of these two indicators on CAFSTB are basically consistent with the previous empirical results. This finding further validates the robustness and explanatory power of the model.

**Reviewer point #4:** *Multicollinearity should be carefully considered in author’s VAR model since it is highly possible that there is multilinear among variables on level of RMB internationalization.*

**Author response #4:** I would like to express my sincere gratitude for your valuable feedback on my manuscript. Meanwhile, I fully understand the questions you raised and respect your rigorous academic vision. However, based on reviewing more relevant literature, I consider that the problem of multicollinearity may not need to be taken too seriously in VAR models, but more attention should be paid to the impulse response analysis and variance decomposition results..

According to Sims[1], Toda[2], and Lütkepohl’s[3] theory, in practical applications, the VAR (vector autoregressive) model usually does not need to worry too much about the problem of multicollinearity. This is mainly because the VAR model's own characteristics and modeling methods prevent multicollinearity from having a serious impact on it. Here are some explanations from these scholars on this issue:

(1) The variables in the VAR model are usually endogenous variables, and there is mutual influence between them, unlike the causal relationship in the traditional panel regression model. This endogeneity characteristic makes the VAR model less sensitive to multicollinearity.

(2) The VAR model assumes that variables are orthogonal, that is, there is no collinearity between them. This assumption enables the VAR model to better handle the relationship between variables. Even if there is a certain degree of correlation, it will not cause serious problems to the model.

(3) The VAR model is a dynamic model that takes into account the lag effect of time series data. Therefore, during the modeling process, lag terms are introduced to capture the dynamic relationship between variables, thereby reducing the impact of multicollinearity.

In addition, after reviewing other scholars’ research using VAR models, I found that there was no content or chapter that mentioned the multicollinearity. You may refer to the following literature. Although VAR models are less sensitive to multicollinearity than traditional regression models, some details still need to be paid attention to during the modeling process to ensure the robustness and accuracy of the model. For example, we should scientifically select the lag order, add appropriate control variables, improve the quality of time series data, etc. I would like to express my sincere thanks again for your suggestions. I will keep paying attention to VAR model methodology research, and carefully consider this problem in future study.

**References**

[1] Sims CA. Macroeconomics and reality. Econometrica: journal of the Econometric Society. 1980:1-48. <https://doi.org/10.2307/1912017>
[2] Toda HY, Yamamoto T. Statistical inference in vector autoregressions with possibly integrated processes. Journal of econometrics. 1995;66(1-2): 225-250. [https://doi.org/10.1016/0304-4076(94)01616-8](https://doi.org/10.1016/0304-4076(94)01616-8)
[3] Lütkepohl H. New introduction to multiple time series analysis. Springer Science & Business Media. 2005. <https://doi.org/10.1007/978-3-540-27752-1>
[4] Ueda RM, Souza AM, Menezes RMCP. How macroeconomic variables affect admission and dismissal in the Brazilian electro-electronic sector: A VAR-based model and cluster analysis. Physica A: Statistical Mechanics and its Applications. 2020;557: 124872. [https://doi.org/10.1016/j.physa.2020.124872](https://doi.org/10.1016/j.physa.2020.124872)
[5] Rodrigues SD, Ueda RM, Barreto AC, Zanini, RR, Souza AM. How atmospheric pollutants impact the development of chronic obstructive pulmonary disease and lung cancer: A var-based model. Environmental Pollution.2021;275: 116622. [https://doi.org/10.1016/j.envpol.2021.116622](https://doi.org/10.1016/j.envpol.2021.116622)
[6] Park C, Chung M, Lee S. The effects of oil price on regional economies with different production structures: A case study from Korea using a structural VAR model. Energy Policy.2011;39(12): 8185-8195. [https://doi.org/10.1016/j.enpol.2011.10.018](https://doi.org/10.1016/j.enpol.2011.10.018)
[7] Brissimis SN, Magginas NS. Forward-looking information in VAR models and the price puzzle. Journal of Monetary Economics. 2006;53(6): 1225-1234. [https://doi.org/10.1016/j.jmoneco.2005.05.014](https://doi.org/10.1016/j.jmoneco.2005.05.014)
[8] Liu S, Huang M, Yushui LI. Chinese agricultural insurance development in a VAR model. Procedia Computer Science. 2022;202: 399-407. [https://doi.org/10.1016/j.procs.2022.04.056](https://doi.org/10.1016/j.procs.2022.04.056)

**Reviewer point #5:** *Minor errors: 4.1 and 4.2 titles are the same; English writing could be improved by academic editing.*

**Author response #5:** I sincerely appreciate your advice on my manuscript. I have carefully checked the minor errors you mentioned and made corrections (on page 23). In response to editor’s insightful recommendations, I have taken the initiative to utilize the esteemed Editage(www.editage.com)’s Language Editing services. Furthermore, I have diligently revised the manuscript, paying particular attention to rectifying any grammatical errors and enhancing the overall quality of the language used.

I express my sincere gratitude for your valuable and perceptive comments.

**Response to Reviewer 2:**

**Reviewer point #1:** *The introduction need to be revised. The introduction gives too much background and requires only a concise paragraph. The author is too slow to get to the point and it detracts from the reading experience. The introduction of the article places too much emphasis the importance of the financial industry without discussing why the study was conducted. The introduction should explain the background meaning of this paper. Start with your research question identify your subject and discuss your motivation. Words like gap and first time need to be used with caution in the elaboration of marginal contribution.*

**Author response #1:** I would like to express my gratitude for your invaluable feedback, which has significantly enhanced the quality of my research. In light of your suggestions, I acknowledge that the information could benefit from greater conciseness. Therefore, I deleted too much discussion of the importance of the financial industry and other redundant statements. Based on your suggestion, I introduce the research background of this article in the first paragraph. The second paragraph emphasizes the research theme of this article, RMB internationalization. In the third paragraph, I elaborate on the research motivation and research process. Finally, in the fourth paragraph, I introduced the marginal contribution of this article and modified some overly absolute terms like “gap”, “first time” and so on. (on page 2-5)

**Reviewer point #2:** *The literature review need to be revised. You should discuss tell what is the new thing we learn from your paper that we previously didn’t know about. Furthermore, the literature review needs to be more adequate. It is suggested that the author refer to the following literatures and systematically reviewed them.*

*Li, Z., Mo, B., & Nie, H. (2023). Time and frequency dynamic connectedness between cryptocurrencies and financial assets in China. International Review of Economics & Finance, 86, 46-57.*

*Yang, C., Chen, L., & Mo, B. (2023). The spillover effect of international monetary policy on China’s financial market. Quantitative Finance and Economics, 7(4), 508-537.*

*Lin, Y. X., Chen, X., & Lan, H. Y. (2023). Analysis and prediction of American economy under different government policy based on stepwise regression and support vector machine modelling. Data Science in Finance and Economics, 3(1), 1-13.*

**Author response #2:** I acknowledge and appreciate your insightful feedback on the literature review section of the manuscript. I would like to inform you that I have properly considered your feedback and in the last paragraph of this section I focus on the shortcomings found in existing research and the implications for how this article can improve these issues. Meanwhile, in order to make the content and logic of this article more adequate and complete, I extensively reviewed relevant relevant literature and cited them in different parts of the new manuscript[1][2][3][4][5][6][7][8]. In addition, I am deeply grateful for the literature references you provided for this study and I believe they were helpful in improving the quality of the content of this article.

Through an in-depth study of the first article[9], I discovered its relevance in facilitating readers' understanding of the economic impact of changes in government policy. This is closely consistent with the research context discussed in this article. Therefore, I have included this reference in the introduction (on page 4) to provide readers with an enhanced understanding of the topic.

The second article[10] analyzes the impact of global financial integration and the monetary policies of the United States, the European Union, and Japan on China's financial market, which is highly relevant to the research focus of this article. Therefore, I cite this source in the “the economic effect of RMB internationalization” section of my literature review (on page 6). Additionally, I use insights from this reference to refine and enhance my literature review.

The third article[11] explores the dynamic time-frequency connection between China's cryptocurrency and traditional financial assets, which is highly relevant to this article's theoretical analysis on the internationalization of China's financial assets and the opening of financial markets. Therefore, I cite this source in my theoretical analysis section (on page 14). Insights from this literature make this study more logical and convincing in explaining why the international nature of RMB financial assets should be promoted.

**References**

[1] Roukanas S, Karakostas E. The Financial Services Exports of China and the Role of RMB. In Economic and Financial Challenges for Balkan and Eastern European Countries: Proceedings of the 10th International Conference on the Economies of the Balkan and Eastern European Countries in the Changing World (EBEEC) in Warsaw. 2020;Poland 2018: 73-87. Springer International Publishing. <https://doi.org/10.1007/978-3-030-39927-6_5>
[2] Sims CA. Macroeconomics and reality. Econometrica: journal of the Econometric Society. 1980:1-48. <https://doi.org/10.2307/1912017>
[3] Wang LR, Shen YT, Shen XR, Xie CJ. Research on the international competitiveness of China's financial services trade based on VAR model. Economic Issues. 2016;07: 55-58+90. [https://doi.org/10.16011/j.cnki.jjwt.2016.07 .011](https://kns.cnki.net/kcms2/article/abstract?v=M7N75Hb03FVygxzN7MuEYXcOGqU5CNgavctyh1OjuKUJo7D4FVCJ7vPPq-jUqbK3pFbzD9TE-9EGqLGXUi6GKeZEE7RjI5e3ZKIav9w6dAy_GBJ77qvcF8Qm-EDMMSSODqv8g2asuImTeTTFTvLacQ==&uniplatform=NZKPT&language=CHS)
[4] [Li Y. Non-international currencies, currency internationalization and capital account convertibility. Financial Research. 2003;08: 104-111.](https://kns.cnki.net/kcms2/article/abstract?v=M7N75Hb03FV5jx1E6AlVUHBzPred3n_I_x7zrOpKQA9hY7-i_j_LeALagmZkIrQAf2pDWK2QfzttRNPr0UcNcfdPUgKx6yREZbVSPIW_Lm5s9lE8Lw7U2yPoAxCha4XgCJLQiLHnEZw=&uniplatform=NZKPT&language=CHS)
[5] Khoury AC, Sawides A. Openness in services trade and economic growth[J]. Economics Letters, 2006;92(2): 277-283. [https://doi.org/10.1016/j.econlet.2006.03.003](https://doi.org/10.1016/j.econlet.2006.03.003)
[6] [Dai X, Zhang E. Do changes in the RMB exchange rate affect the growth of China's service exports? Financial Research 2014;11: 22-34.](https://kns.cnki.net/kcms2/article/abstract?v=M7N75Hb03FXkIg-hJEMZ0vmaVBCUcfRLlbq4tppNSQ0ZpcQb2Y0ULVerzAHx0_EwbuoATS91q5nCJTRytLvjbOymOd6SFfW2iztdCzPmEb4zMi1u2qDxYtB4WmoqKrXGHGI_3ZfqWKbQlbS9LbvMjQ==&uniplatform=NZKPT&language=CHS)
[7] Toda HY, Yamamoto T. Statistical inference in vector autoregressions with possibly integrated processes. Journal of econometrics. 1995;66(1-2): 225-250. [https://doi.org/10.1016/0304-4076(94)01616-8](https://doi.org/10.1016/0304-4076(94)01616-8)
[8] [Xiang Yuanxin & Wang Xuemei. Research on the impact of currency internationalization on the competitiveness of financial services trade. Western Forum. 2022;04:41-54.](https://kns.cnki.net/kcms2/article/abstract?v=M7N75Hb03FX4ON-Xgymo4KQ6E7Pf-0PK-UrHtfabjOcTPK_db4PsZ4KJOq6Z4xbuBDiUFiveca3SSYO66pNTeb0oFJUDVNaWB02o5LE9wo_8Un11ztgTkqX-j2WBWuxT55vtBREEqP-8fInPp6-NPw==&uniplatform=NZKPT&language=CHS)
[9] Lin YX, Chen X, Lan HY. Analysis and prediction of American economy under different government policy based on stepwise regression and support vector machine modelling. Data Science in Finance and Economics. 2023;3(1): 1-13. <https://doi.org/10.3934/DSFE.2023001>
[10] Yang C, Chen L, Mo B. The spillover effect of international monetary policy on China’s financial market. Quantitative Finance and Economics. 2023;7(4): 508-537. <https://doi.org/10.3934/QFE.2023026>
[11] Li Z, Mo B, Nie H. Time and frequency dynamic connectedness between cryptocurrencies and financial assets in China. International Review of Economics & Finance. 2023;86: 46-57. [https://doi.org/10.1016/j.iref.2023.01.015](https://doi.org/10.1016/j.iref.2023.01.015)

**Reviewer point #3:** *The author has no relevant explanation for the reason for using the model. Furthermore, the paper lacks details on the implementation of the VAR model. It would be helpful to provide information on the lag length selection, model specification, and estimation techniques.*

**Author response #3:** I would like to express my gratitude for your valuable feedback, which greatly improved the quality of my research. After careful consideration of your comments, I revised and improved the original manuscript. Specifically, after reviewing more literature, I added the specific implementation process of the VAR model in the model design section (on pages 16-18), and explained in detail why the VAR model is used in this article and the advantages of using this model. Furthermore, in the section "Determination of optimal lag order for VAR model" (on pages 23-24), the new manuscript gives the computational logic of the relevant criteria used when choosing the lag length. Based on your suggestions, I firmly believe that the addition of the above content can make the academic thinking and writing logic of this study more rigorous and scientific.

**Reviewer point #4:** *The paper does not discuss the limitations of the VAR model and potential sources of endogeneity or omitted variable bias.*

**Author response #4:** I sincerely appreciate your insightful feedback. In response to this feedback, I have made significant revisions, including a discussion of the study's limitations. Although the VAR model has obvious advantages for this study, as you pointed out, the model still has many limitations and shortcomings. Therefore, this study has added the sixth part "Limitations and future research directions", which profoundly explains the shortcomings of this study and the VAR model, and puts forward my opinions on how future research should be improved.

In addition, in order to alleviate the possible endogeneity problem in the model caused by omitted variables, this paper extensively reviewed more studies. Considering data availability and the loss of a certain degree of freedom due to the length of the lag period in the VAR system, three control variables will be selected and added to the model (on pages 20-21). This approach can alleviate the endogeneity problem caused by omitted variables to a certain extent. Furthermore, the VAR model aims to describe the dynamic relationship between endogenous variables, and its basic assumption is that all variables are endogenous. That is, they are determined by internal factors of the system and have intrinsic correlation and influence with each other. Therefore, in the VAR model, all variables are considered endogenous variables. However, by choosing an appropriate lag order, we can better capture the dynamic relationship between variables, thereby reducing problems caused by endogeneity. Therefore, after adding new control variables, the new manuscript re-selects the appropriate lag order according to relevant criteria.

**Reviewer point #5:** *This paper based on the time series data of China-US financial services trade from 2010 to 2021. However, the author said in the introduction that China has taken a series of measures to promote the internationalization of RMB since 2008. So why doesn’t the author use earlier data to compare the differences before and after the internationalization of RMB?*

**Author response #5:** I would like to express my sincere gratitude for your valuable and insightful comments and understand your doubts about the selection of data time span. This article chooses to use data from 2010 to 2021 for analysis due to the following considerations:

(1) Data reliability and integrity. After the financial crisis broke out in 2008, the Chinese government began to discuss promoting the use of RMB in international trade and finance. In 2009, it formally proposed the "RMB internationalization" plan, aiming to improve its international status and influence. The "RMB Internationalization Index Report" released by China Renmin University in 2010 is the first official document on the measurement of RMB internationalization. Therefore, after China took a series of measures to promote the internationalization of the RMB in 2008, the recording and reporting of relevant data will take a certain amount of time to be standardized and detailed. In contrast, there may be uncertainties in the quality and completeness of earlier data.

(2) Eliminate the impact of emergencies. The global financial crisis in 2008-2009 and a series of policy measures taken by China may have an important impact on the data and results. Therefore, selecting data from 2010 and after for analysis can better eliminate the impact of emergencies on RMB internationalization and financial services trade.

Although choosing to use earlier data for comparative analysis may help provide a more comprehensive understanding of the differences before and after RMB internationalization, analysis based on data from 2010 to 2021 can still provide useful conclusions and insights. In terms of research design and data selection, this article comprehensively considers factors such as research questions, data reliability, and research focus to ensure the scientificity and reliability of the research.

**Reviewer point #6:** *The economic explanation of the empirical results is insufficient. In this paper, each empirical result is only to explain whether the relevant test statistics are significant or not, but the explanation of economics is not enough. It is suggested that the graph and table involving the empirical results should be combined for economic explanation. In fact, statistical significance can only explain the "what", but does not explain the "why", that is, the paper needs to explain the "why" based on the econometric analysis.*

**Author response #6:** I would like to express my gratitude for your valuable comments, which greatly improved the quality of my research. In the VAR model analysis of this article, the tests conducted in 4.1-4.5 are to illustrate the reliability of the model. The impulse response functions, variance decomposition results and robustness tests conducted in 4.6-4.8 are important outputs of the empirical results, and they can provide important information about the internal dynamic relationships of the economic system and the impact transmission mechanism. Therefore, based on your suggestions, this article combines more economic logic and social background to explain the analysis results in chapters 4.6-4.8 (On pages 26-33). These modifications provide a more scientific economic explanation for the empirical results of this article.

Once again, I sincerely appreciate your insightful comments and your role in improving my manuscript.

I would like to express my sincere gratitude for your insightful comments and your valuable contribution towards enhancing the quality of my manuscript. I have made diligent efforts to enhance my manuscript and have implemented several changes accordingly. It is important to note that these modifications have been made without altering the underlying content of my manuscript.

I extend my heartfelt gratitude to the editor and the two reviewers for their dedicated and diligent efforts in evaluating my manuscript. I earnestly hope that the revisions I have made will meet your expectations and receive your approval.

Once again, thank you very much for your valuable comments and suggestions.

Yours faithfully

Yufei Lei
