# Supplementary material for: Impact of RMB internationalization on China’s competitiveness in financial services trade based on the VAR model: Evidence from China-US
Source: PLoS One. 2024 Jun 4;19(6):e0302845. doi: 10.1371/journal.pone.0302845 (PMC11149869; doi:10.1371/journal.pone.0302845)
Supplement: Attachment — Submitted filename: Comments on PONE-D-23-42358 (second round).docx [file pone.0302845.s004.docx]

**Comments on**

**Impact of RMB Internationalization on China’s Competitiveness in Financial Services Trade based on VAR Model: Evidence from China-US**

Second Round

Recommendation: Accept

Author carefully revised manuscript according to reviewers’ comments and suggestions. Response letter is written well to reply to questions proposed by reviewers one by on.
